# Prescriptions for selective cyclooxygenase-2 inhibitors, non-selective non-steroidal anti-inflammatory drugs, and risk of breast cancer in a population-based case-control study

**DOI:** 10.1186/bcr2482

**Published:** 2010-03-01

**Authors:** Deirdre P Cronin-Fenton, Lars Pedersen, Timothy L Lash, Søren Friis, John A Baron, Henrik T Sørensen

**Affiliations:** 1 Department of Clinical Epidemiology, Aarhus University Hospital, Olof Palmes Alle 43-45, 8200 Aarhus N, Denmark; 2 Department of Epidemiology, School of Public Health, Boston University, 715 Albany Street, TE3, Boston, MA 02118, USA; 3 Institute of Cancer Epidemiology, The Danish Cancer Society, Strandboulevarden 49, 2100 Copenhagen Ø, Denmark; 4 Departments of Community and Family Medicine and Medicine and the Norris Cotton Cancer Center, Dartmouth Hitchcock Medical Center, Lebanon, NH, USA

## Abstract

**Introduction:**

Non-steroidal anti-inflammatory drugs (NSAIDs) prevent the growth of mammary tumours in animal models. Two population-based case-control studies suggest a reduced risk of breast cancer associated with selective cyclooxygenase-2 (sCox-2) inhibitor use, but data regarding the association between breast cancer occurrence and use of non-selective NSAIDs are conflicting.

**Methods:**

We conducted a population-based case-control study using Danish healthcare databases to examine if use of NSAIDs, including sCox-2 inhibitors, was associated with a reduced risk of breast cancer. We included 8,195 incident breast cancer cases diagnosed in 1991 through 2006 and 81,950 population controls.

**Results:**

Overall, we found no reduced breast cancer risk in ever users (>2 prescriptions) of sCox-2 inhibitors (odds ratio (OR) = 1.08, 95% confidence interval (95% CI) = 0.99, 1.18), aspirin (OR = 0.98, 95% CI = 0.90-1.07), or non-selective NSAIDs OR = 1.04, (95% CI = 0.98, 1.10)). Recent use (>2 prescriptions within two years of index date) of sCox-2 inhibitors, aspirin, or non-selective NSAIDs was likewise not associated with breast cancer risk (Ors = 1.06 (95% CI = 0.96, 1.18), 0.96 (95% CI = 0.87, 1.06) and 0.99 (95% CI = 0.85, 1.16), respectively). Risk estimates by duration (<10, 10 to 15, 15+ years) or intensity (low/medium/high) of NSAID use were also close to unity. Regardless of intensity, shorter or long-term NSAID use was not significantly associated with breast cancer risk.

**Conclusions:**

Overall, we found no compelling evidence of a reduced risk of breast cancer associated with use of sCox-2 inhibitors, aspirin, or non-selective NSAIDs.

## Introduction

Non-steroidal anti-inflammatory drugs (NSAIDs) are inversely associated with the risk of colorectal and other gastrointestinal cancers (for example, stomach and oesophageal cancer) [1-5]. The protective effect of NSAIDs against these cancers has prompted studies on breast cancer prevention by NSAIDs.

Research on human cell lines and animal models indicates a role for cyclooxygenase-2 (Cox-2) in breast carcinogenesis [6], which suggests that selective Cox-2 (sCox-2) inhibitors and NSAIDs may prevent the growth of mammary tumours [7-14]. Some NSAIDs are more potent against Cox-1 (for example, aspirin), others have greater affinity for Cox-2 (sCox-2 inhibitors), while others are relatively non-selective (for example, naproxen) [15]. Cox-1 is ubiquitously and constitutively expressed, while Cox-2 is induced in response to stimuli such as cytokines [16] and is overexpressed in approximately 40% of human breast tumours [17,18]. NSAIDs may exert a protective effect against breast cancer by inhibiting Cox-2 and, in turn, reducing the level of prostaglandins, oestrogens and/or prolactin [5,15,19-24].

Results from epidemiological studies of breast cancer, however, are conflicting [25,26]. To date, five meta-analyses have indicated chemopreventive effects of aspirin or NSAIDs against breast cancer [1,27-30]. Some cohort and case-control studies have reported no reduced risk of breast cancer either from use of non-aspirin NSAIDs (NA-NSAIDs) [31-38] or aspirin [7,26,31,35-45]. Others have suggested a reduced risk associated with NA-NSAIDs [8,46-53] and aspirin [8,32,46-52,54-59], albeit less marked than that observed for colorectal cancer (approximately 30% versus approximately 50% reduction) [58,60,61]. The conflicting evidence may be attributable to a combination of factors including poor precision and chance variation [39,43,47,48,58], low response rates with possible selection bias [7,51], short follow-up time following prescription [36,47,59], limited exposure data [27,57], or failure to distinguish between different NSAIDs subclasses [33,34,36,37,53]. Only two, case-control, studies have investigated the association of newer sCox-2 inhibitors and breast cancer occurrence; both found decreased breast cancer risks [47,59], but only one study adjusted for previous use of NSAIDs in the analyses.

To answer some of the research gaps in the epidemiological literature, we conducted a large population-based case-control study nested within a source population with prospectively collected prescription data to examine the association between use of sCox-2 inhibitors, aspirin, or non-selective NA-NSAIDs and the risk of breast cancer occurrence.

## Materials and methods

This study was approved by the Danish Registry Board, reference #2004-41-4693.

### Source population

We conducted this nested population-based case-control study among the residents of North Jutland and Aarhus counties, Denmark, which together have a total population of 1.15 million inhabitants [62]. The Danish National Health Service provides free tax-supported healthcare to all residents of the country and refunds part of patient expenditures on most prescribed drugs, including aspirin, non-selective NA-NSAIDs, and sCox-2 inhibitors. Selective Cox-2 inhibitors include the older sCox-2 inhibitors (for example, meloxicam), and newer sCox-2 inhibitors (for example, rofecoxib, celecoxib, and so on), which became available in Denmark in 1999. Rofecoxib was withdrawn in 2004, and use of the other newer sCox-2 inhibitors including celecoxib, has declined (see [63] for types of NSAIDs and sCox-2 inhibitors and package sizes available in Denmark).

All health-related services are registered to individual patients by use of their civil personal registration (CPR) number, assigned to all Danish citizens since 1968 by the Danish Civil Registration System. This number encodes gender and date of birth [64] and allows accurate linkage between population-based registries, including the National Registry of Patients and the prescription databases [65].

#### Breast cancer cases

Healthcare data from the two counties have been merged into a research database at Aarhus University [65-67]. This database includes all non-psychiatric hospital admissions since 1977 and out-patient hospital clinic visits since 1994 among the residents of the counties. Information is recorded immediately after discharge or outpatient visit and includes CPR number, dates of admission and discharge, and up to 20 diagnoses coded by International Classification of Diseases (ICD) codes (versions 8^th ^(1977 to 2003) and 10^th ^(1994 on)) [66]. Using these databases, we identified all female patients who had a first diagnosis of breast cancer (ICD-8 codes 174.01, 174.02, 174.08 and 174.09; ICD-10 codes C50.0-50.6, C50.8 and C50.9) between 1 January 1991 and 31 December 2006 in North Jutland County and between 1 January 1998 and 31 December 2006 in Aarhus County. To ensure that we had at least minimal prescription data for each case (see below), we excluded 127 cases who had not been resident in the study area for at least two years, leaving a total of 8,195 breast cancer cases.

#### Population controls

We used the Civil Registration System [64,68] to select 10 population controls for each case, matched on birth year and county of residence, using risk-set sampling (that is, the controls had to be alive and at risk of breast cancer at the time the corresponding case was diagnosed (index date)). We included a total of 81,950 population controls, sampling only among individuals who were resident in the study areas at least two years before index date.

### Data collection

#### Prescription data

All pharmacies in both counties are equipped with computerised accounting systems that record a customer's CPR number, type and quantity of medication (including tablet and package sizes), and information on the drug according to the Anatomical Therapeutic Classification (ATC) (World Health Organization Collaborating Centre for Drug Statistics Methodology, 2001). Healthcare prescription data for refundable drugs have been transferred electronically to a research database at Aarhus University; in North Jutland County since 1989, and in Aarhus County since 1996. We identified prescriptions for the following sCox-2 inhibitors: newer sCox-2 inhibitors (celecoxib M01AH01, rofecoxib M01AH, valdecoxib M01AH03, etoricoxib M01AH05), and older sCox-2 inhibitors (lornoxicam M01AC05, diclofenac M01AB05, meloxicam M01AC06). We grouped both newer and older sCox-2 inhibitors together. Aspirin use was classified as low dose (B01AC06 and N02BA01 in tablet sizes of 75, 100 or 150 mg) and high dose (N02BA51 and N02BA01 in tablet size 500 mg). We grouped the other non-selective NA-NSAIDs (remaining codes within M01A) into a separate category. We also retrieved prescriptions for post-menopausal hormone replacement therapy (G03C, G03D, G03F, G03HB01), since use of hormone replacement therapy increases the risk of breast cancer [69-71] and women who adhere to one medication may also be more likely to adhere to other prescription medications, such as NSAIDs [72]. Although acetaminophen is mainly obtained over the counter in Denmark, we also investigated the impact of prescribed acetaminophen (N02BE51) use on breast cancer risk as a comparison analysis [73].

#### Comorbidity data

We retrieved information on a hospital history of rheumatoid arthritis and migraine from the research database as proxies for chronic use of over-the-counter NA-NSAIDs and aspirin. Comorbid diseases before the diagnosis/index date were identified using the hospital discharge registries of each county and were summarized using the Charlson Index [74]. The index comprises 19 conditions, each weighted according to its potential to influence mortality. We included both inpatient and outpatient data and used Deyo's adaptation of the Charlson comorbidity score for ICD-9-CM diagnoses [75]. We obtained information on history of heart disease (myocardial infarction, congestive heart failure, peripheral vascular disease) from the Danish National Registry of Patients (DNRP).

### Analytic variables

We excluded use of all NSAIDs within a year of diagnosis to reduce any potential effect that subclinical disease could have on NSAIDs and sCox-2 inhibitor use (that is, reverse causation). We categorised NSAID use according to the number of prescriptions filled by each patient. *Ever users *were defined as women who had more than two prescriptions and *never/rare users *were women who used less than or equal to two prescriptions. Our data showed that the average length of a prescription was 30 days.

We examined temporality of NSAID use by dividing *ever users *into recent and former users. Recent users were those who had three or more prescriptions within two years of index date (that is, between one and two years before index date). Former users were those who had less than three prescriptions within that time period but more than two prescriptions (as defined by *ever *use) during the entire period of observation.

We examined whether breast cancer risk was associated with the intensity of NSAID use. The intensity of use was defined as low (less than 25%), medium (25 to 50%) or high (greater than 50%), according to the number of days of prescription coverage divided by the total duration of use in days. The duration was defined as the number of days from the date of a first prescription to the date of a last prescription plus the duration of the last prescription. We divided duration of NSAID use into long-term (10 to less than or equal to15 years, and greater than 15 years) and shorter-term (less than 10 years) use. In most analyses, we considered sCox-2 inhibitors and non-selective NSAIDs separately. However, to examine intensity and duration of NSAID use, we grouped all NSAIDs together and included only women with at least 10 years of prescription history, that is, cases from North Jutland County diagnosed between 1999 and 2006, and from Aarhus County diagnosed in 2006, and their corresponding controls. This approach ensured that women who were long-term users were not misclassified as shorter-term due to a limited prescription history.

The cut points for medication exposures were determined based on methods developed by Robertson et al. [76]. Robertson et al used the number of pills of proton pump inhibitors to assess exposure. We modified the methods used in the Robertson paper and used prescriptions rather than number of pills to account for the variation in NSAID pill strengths.

### Statistical analyses

In all analyses, we used conditional logistic regression to compute odds ratios (ORs) and their associated 95% confidence intervals (95% CI) adjusting for a history of migraine and rheumatoid arthritis, and ever/never hormone replacement therapy use. We completed additional analyses adjusting for the Charlson comorbidity index (also as a proxy for chronic NSAID use) and separate analyses adjusting for history of heart diseases, but these had no appreciable effect on the risk estimates and were therefore dropped from the final models. In each analysis, we used never or rare use (less than or equal to two prescriptions in total) as the reference group. Given the risk set sampling of controls, the ORs were unbiased estimates of the incidence rate ratios (IRRs) in the underlying population. Analyses were performed using SAS version 9.13 (SAS Institute Inc., Cary, North Carolina, USA).

## Results

Characteristics of the (8,195) breast cancer cases and (81,950) population controls are presented in Table 1. Mean age at index date was 62.1 years. Slightly more cases than controls had a hospital history of migraine. As expected, a higher proportion of cases than controls had ever used hormone replacement therapy (23.8% versus 19.3%, *P *<0.001). More cases than controls had ever used sCox-2 inhibitors (8.6 versus 7.7%, *P *= 0.007) and/or NA-NSAIDs (22.4 versus 21.2%, *P *= 0.02).

**Table 1:** Frequency distribution of cases and matched population controls (number and percentage)

Characteristic		Cases*		Controls*	
	*P-value*	N = 8,195	%	N = 81,950	%
**Selective Cox-2 Inhibitors**	*0.007*				
No		7,362	89.8	74,360	90.7
Yes		701	8.6	6,314	7.7
**Non-selective Non-aspirin**	*0.02*				
**Non-Steroidal Anti- Inflammatory Drugs**					
No		6,361	77.6	64,549	78.8
Yes		1,834	22.4	17,401	21.2
**Low Dose Aspirin**	*0.93*				
No		7,414	90.5	74,115	90.4
Yes		781	9.5	7,835	9.6
**High Dose Aspirin**	*0.28*				
No		8,168	99.7	81,733	99.7
Yes		27	0.3	217	0.3
**Acetaminophen**	*0.64*				
No		7,317	89.3	73,033	89.1
Yes		878	10.7	8,917	10.9
**Rheumatoid Arthritis**	*0.70*				
No		7,640	93.2	76,490	93.3
Yes		555	6.8	5,460	6.7
**Migraine**	*0.002*				
No		7,797	95.1	78,571	95.9
Yes		398	4.9	3,379	4.1
**Hormone Replacement Therapy**	*<0.001*				
Never		6,248	76.2	66,132	80.7
Ever		1,947	23.8	15,818	19.3

*Matched on county of residence and birth year

Overall, we observed no reduced risk of breast cancer associated with ever versus never/rare use of sCox-2 inhibitors (OR = 1.08, 95% CI = 0.99 to 1.18), non-selective NA-NSAIDs (OR = 1.04, 95% CI = 0.98 to 1.10), or aspirin (OR = 0.98, 95% CI = 0.90 to 1.07) (Table 2). The timing of non-selective NA-NSAID or sCox-2 inhibitor use also did not influence breast cancer risk. Compared with never/rare use, recent use (OR = 0.98, 95% CI = 0.84 to 1.15) and former use (OR = 1.12, 95% CI = 1.02 to 1.24) of sCox-2 inhibitors were not associated with breast cancer occurrence. The corresponding risk estimates for use of non-selective NA-NSAIDs were 1.06 (95% CI = 0.95 to 1.17) and 1.03 (95% CI = 0.97 to 1.10), and for use of aspirin were 0.96 (95% CI = 0.87 to 1.06) and 1.02 (95% CI = 0.89 to 1.17). Selective Cox-2 inhibitor use was not associated with breast cancer occurrence among individuals with (OR = 1.12, 95% CI = 0.86 to 1.44) or without (OR = 1.07, 95% CI = 0.98 to 1.17) a history of NSAID use before beginning sCox-2 inhibitor use. There was no evidence of a reduced risk of breast cancer among women who used sCox-2 inhibitors only (ever use OR = 1.09, 95% CI = 0.98 to 1.22; recent use OR = 0.99, 95% CI = 0.80 to 1.23; former use OR = 1.13, 95% CI = 1.00 to 1.29). We found no association of prescriptions for acetaminophen with breast cancer occurrence (data not shown).

**Table 2:** Temporality of non-steroidal anti-inflammatory drug use and odds ratio of breast cancer

Characteristics	Cases* (N = 8,195)		Controls* (N = 81,950)		Odds Ratio^	95% Confidence Interval
	N	%	N	%		
**Any NSAID use**						
Ever versus never					1.04	0.99 to 1.10
Never/rare^1^	5,353	65	54,742	67	1.00	
Recent use^2^	1,147	14	11,236	14	1.00	0.93 to 1.08
Former use^3^	1,695	21	15,972	20	1.02	0.96 to 1.09
						
**Selective Cox-2 Inhibitors**						
Ever versus never					1.08	0.99 to 1.18
Never/rare^1^	6,361	78	64,549	79	1.00	
Recent use^2^	442	5	4,105	5	0.98	0.84 to 1.15
Former use^3^	1,392	17	13,296	16	1.12	1.02 to 1.24
						
**Non-selective Non-aspirin**						
**Non-Steroidal Anti-Inflammatory Drugs**						
Ever versus never					1.04	0.98 to 1.10
Never/rare^1^	7,494	91	75,636	92	1.00	
Recent use^2^	184	2	1,812	2	1.06	0.95 to 1.17
Former use^3^	517	6	4,502	6	1.03	0.97 to 1.10
						
**Aspirin**						
Ever versus never						
Never/rare^1^	7,396	90	73,947	90	0.98	0.90 to 1.07
Recent use^2^	548	7	5,596	7	0.96	0.87 to 1.06
Former use^3^	251	3	2,407	3	1.02	0.89 to 1.17

* Matched on county of residence and birth year. ^Analyses adjusted for use of hormone replacement therapy, rheumatoid arthritis and migraine. ^1 ^Never/rare use: <3 prescriptions in total. ^2 ^Recent use: >2 prescriptions within two years of diagnosis. ^3 ^Former use, </= 2 prescriptions within two years of diagnosis. NSAIDs, non-steroidal anti-inflammatory drugs.

In analyses that included all NSAID use (selective, non-selective and aspirin), we found no protective effect against breast cancer in shorter-term or long-term users, regardless of the intensity of use (Table 3). We carried out additional analyses stratifying by drug type (see Additional file 1).

**Table 3:** Duration and intensity of NSAID¤ use among women with ≥ 10 years of prescription history

Characteristics	Cases*		Controls*		Odds Ratio	95% Confidence Interval
	N	%	N	%		
Never/rare	1,188	50	12,714	53	1.00	
						
Shorter-term <10 years:						
Low Intensity†	317	13	2,997	13	1.09	0.96 to 1.25
Medium Intensity‡	210	9	2,078	9	1.03	0.88 to 1.20
High Intensity^+^	332	14	2,961	12	1.16	1.01 to 1.33
						
Long-term 10 to </= 15 years:						
Low Intensity†	151	6	1,301	5	1.18	0.98 to 1.42
Medium Intensity‡	81	3	773	3	1.06	0.83 to 1.35
High Intensity^+^	88	4	971	4	0.90	0.71 to 1.13
						
Long-term >15 years:						
Low Intensity†	6	0.3	76	0.3	0.80	0.35 to 1.85
Medium Intensity‡	16	0.7	74	0.3	2.30	1.30 to 4.06
High Intensity^+^	10	0.4	96	0.4	1.01	0.52 to 1.97

¤(sCox-2 inhibitors, aspirin, and non-aspirin non-selective NSAID). * Matched on county of residence and birth year. ^Analyses adjusted for use of hormone replacement therapy, history of rheumatoid arthritis and migraine. † Low intensity was prescription use <25% of duration. ‡ Medium intensity was prescription use >25% but <50% of duration. ^+ ^High intensity was prescription use >50% of duration.

## Discussion

In this population-based case-control study, we found no substantial association between risk of breast cancer and use of NSAIDs, whether aspirin, non-selective or Cox-2 selective NSAIDs.

Our results are inconsistent with those of a case-control study by Sharpe *et al*. [53] based on similar prospectively-collected prescription data. Their study reported a dose-dependent reduced risk of up to 24% for breast cancer among short-term (two to five years) users of NSAIDs, an association that is not evident in our study. The Sharpe study [53] and a study by Zhang and colleagues [8] investigated the impact of long-term (over 10 years) use of NSAIDs on breast cancer occurrence. Both papers suggested that long-term use of NSAIDs conferred a modest protective effect. The Zhang study also examined very long-term (over 20 years) use of NSAIDs and found a 38% reduction in risk, but this was not statistically significantly different from estimates for shorter-term use [8]. Our study shows no evidence of a reduced risk of breast cancer associated with either shorter-term or long-term use of NSAIDs.

The only two previous studies to examine the impact of sCox-2 inhibitors on breast cancer risk reported a risk reduction of at least 20% [47,77]. Both studies, however, had important limitations, which should be considered when interpreting their findings. One, a hospital-based case-control study, sampled cases and controls from different base populations; all cases in the cancer hospital were eligible, however controls were selected among women attending the hospital's mammography service, so may not have been representative of the population that gave rise to the cases [47]. The other, a Canadian registry-based study, had accurate exposure data sourced from an insurance database, however it only had a maximum exposure period of three years [59]. As women who were long-term users of NSAIDs may have switched to sCox-2 inhibitors when they became available to avoid any adverse side-effects, a reduced risk of breast cancer in such women could have been attributable to long-term use of NSAIDs rather than sCox-2 inhibitors.

To overcome potential limitations, we included all available data on use of newer sCox-2 inhibitors, the entire period for which they were available on the Danish market [78,79], and longer than in the previous studies [47,59]. We examined the associations of sCox-2 inhibitors with and without consideration of earlier NSAID use, and the risk of breast cancer among women who used sCox-2 inhibitors only, but these additional analyses did not alter the estimates of association. Overall, our findings on ever/never use and recent versus former use of sCox-2 inhibitors do not support a protective effect of sCox-2 inhibitors on breast cancer risk.

Between 5% and 56% of breast cancer patients are estimated to harbour mammogram-detectable carcinomas two to five years before clinical diagnosis [80]. Mammographic screening was not widespread in Denmark during our study period, so it is not clear whether the limited availability of newer sCox-2 inhibitors (approval in 1999 and withdrawal in 2004 due to concern for their cardiovascular toxicity [81-83] enabled sufficient time to intercept the pathway from tumour initiation to detectable disease. Moreover, data from a post-hoc analysis of British aspirin trials reported that five years of aspirin use was associated with a reduced risk of colorectal cancer, but the protective effect required a latent period and first emerged 10 years after treatment initiation [84]. Subjects included in our study may therefore have had insufficient exposure time to enable protection against breast cancer, even if they were diagnosed late in the study period.

The validity of our estimates depends on several factors. First, the use of population-based prescription registries, with a completeness approaching 100% [65], ensured unbiased assessment of exposure data before breast cancer diagnosis and the registry source eliminated potential recall bias. Furthermore, for hormone replacement therapy use, the prescription data are in very good agreement with self-reported use [85]. The use of prescription records may be more accurate than self-reported or in-person interview questionnaire data, particularly when capturing intermittent NSAID use. Second, we had complete follow-up. Using the Danish Cancer Registry as a reference standard, the sensitivity and positive predictive value of a cancer diagnosis in the hospital registry is high [86]. Any misclassification of cases would bias our results to the null because it is unlikely to be associated with NSAID use. In our analyses of duration and intensity of NSAID use, we included only patients with at least 10 years of prescription history, thus bias due to left-censoring of exposure was reduced. Furthermore, we examined the duration and regularity of NSAID use, which are important factors in reducing the risk of colorectal cancer by NSAIDs [7]. The number of cases of breast cancer in the study area determined the sample size of our study and 10 controls were matched for each case. The large size of our study ensured almost 100% power to detect inverse associations with NSAIDs of the size reported earlier [27,59]. Finally, the positive association between hormone replacement therapy use and breast cancer risk observed in our study is consistent with previous research and lends face validity to the null NSAID results.

Our study also had limitations. We were unable to examine the impact of NSAID use on breast cancer risk by specific breast cancer characteristics such as hormone receptor status. Earlier studies suggest that NSAID use can decrease the risk of oestrogen receptor positive tumours in post-menopausal women [87]. However, such a protective effect would have to be counterbalanced by a causal effect in the hormone receptor negative group in order to obtain a null result. As there is no evidence in the literature of such a causal effect in oestrogen receptor negative disease, and given our null result, it is unlikely that our inability to segregate our analyses by hormone receptor status masked any association. We did stratify our analyses by menopausal status but found little change in the risk estimates. We had no data regarding adherence with prescriptions or the use of non-prescription NSAIDs (a consideration likely to be relevant primarily for short-term use). However, our drug exposure assessment was based on redeemed prescriptions, and because patients had to pay a proportion of the drug cost, our estimates are likely to reflect actual use. Furthermore, only low-dose ibuprofen and aspirin were available without prescription in Denmark, the former accounting for approximately 14% of total NSAID use [88]. Although we lacked information on clinical indication for NSAID use, we included an adjustment for migraine and rheumatoid arthritis in our analyses to capture some chronic use of over-the-counter NSAIDs and observed little difference in the risk estimates for history of migraines and/or rheumatoid arthritis between cases and controls. In addition, we evaluated potential confounding by heart disease and the Charlson comorbidity index but found no difference in the risk estimates (see Additional file 1). Finally, due to low numbers it is difficult to draw any conclusions from our analyses on the risk of breast cancer according to duration and intensity of aspirin use (see Additional file 1).

We had no information on family history of breast cancer, body mass index, use of oral contraceptives and life-style factors such as alcohol consumption and dietary fat intake [87]. While these factors may impact on breast cancer risk, it is not clear what association, if any, they would have with NSAIDs or sCox-2 inhibitor use. Furthermore, any confounding by these variables would have to occur even after the adjustments we did make. Therefore, we expect these factors to have little impact on our study findings.

## Conclusions

Our findings in this large population-based setting do not support an association of prescription NSAID use with the risk of breast cancer.

## Abbreviations

95% CI: 95% Confidence Interval; ATC: Anatomical Therapeutic Classification; Cox-2: cyclooxygenase-2; CPR number: civil personal registration number; ICD: International Classification of Diseases; IRRs: incidence rate ratios; NSAIDs: non-steroidal anti-inflammatory drugs; NA-NSAID: non-aspirin NSAID; OR: odds ratio; sCox-2: selective cyclooxygenase-2.

## Competing interests

JAB is a consultant to Bayer Pharmaceuticals, Merck Pharmaceuticals and Pozen Incorporated and holds a US patent regarding the chemopreventive efficacy of aspirin in the large bowel. HTS did not report receiving fees, honoraria, grants or consultancies. The Department of Clinical Epidemiology is, however, involved in studies with funding from various companies as research grants to (and administered by) Aarhus University. None of these studies are related to the present study. All other authors declare no competing interests.

## Authors' contributions

HTS, DCF, SF and LAP conceived the study idea and designed the study. HTS and LAP collected the data. LAP, DCF, JAB, TLL and HTS planned and performed the analyses. DCF reviewed the literature and drafted the manuscript. DCF, TLL, SF, JAB and HTS edited the manuscript.

## Supplementary Material

Additional file 1 **Tables S1-S3**. Table S1: Duration and intensity of non-steroidal anti-inflammatory drug use (sCox-2 inhibitors and non-selective non-aspirin non-steroidal anti-inflammatory drugs) among women with at least 10 years of prescription history and odds ratio of breast cancer. Table S2: Duration and intensity of non-steroidal anti-inflammatory drug use (aspirin and non-selective non-aspirin non-steroidal anti-inflammatory drugs) among women with at least 10 years of prescription history and odds ratio of breast cancer. Table S3: Duration and intensity of aspirin use among women with at least 10 years of prescription history and odds ratio of breast cancer. Click here for file
