# Supplementary material for: Prescriptions for selective cyclooxygenase-2 inhibitors, non-selective non-steroidal anti-inflammatory drugs, and risk of breast cancer in a population-based case-control study
Source: Breast Cancer Res. 2010 Mar 1;12(2):R15. doi: 10.1186/bcr2482 (PMC2879557; doi:10.1186/bcr2482)
Supplement: Additional file 1 — Tables S1-S3. Table S1: Duration and intensity of non-steroidal anti-inflammatory drug use (sCox-2 inhibitors and non-selective non-aspirin non-steroidal anti-inflammatory drugs) among women with at least 10 years of prescription history and odds ratio of breast cancer. Table S2: Duration and intensity of non-steroidal anti-inflammatory drug use (aspirin and non-selective non-aspirin non-steroidal anti-inflammatory drugs) among women with at least 10 years of prescription history and odds ratio of breast cancer. Table S3: Duration and intensity of aspirin use among women with at least 10 years of prescription history and odds ratio of breast cancer. [file bcr2482-S1.docx]

***Appendix Table 1: Duration and intensity of non-steroidal anti-inflammatory drug use (sCox-2 inhibitors and non-selective non-aspirin non-steroidal anti-inflammatory drugs) among women with at least 10 years of prescription history and odds ratio of breast cancer***

| **Characteristics** | **Cases*** | | **Controls*** | | **Odds Ratio^** | | **95% Confidence Interval** |
| --- | --- | --- | --- | --- | --- | --- | --- |
|  | **N** | **%** | **N** | **%** | |  |  |
|  |  |  |  |  | |  |  |
| Never/rare | 1349 | 56 | 14235 | 59 | | 1.00 |  |
|  |  |  |  |  | |  |  |
| Short-term <10 years: |  |  |  |  | |  |  |
| Low Intensity† | 264 | 11 | 2622 | 11 | | 1.02 | 0.89-1.17 |
| Medium Intensity‡ | 233 | 10 | 1901 | 8 | | 1.23 | 1.06-1.42 |
| High Intensity¤ | 272 | 11 | 2650 | 11 | | 1.03 | 0.89-1.18 |
|  |  |  |  |  | |  |  |
| Long-term 10-<=15 years: |  |  |  |  | |  |  |
| Low Intensity† | 133 | 6 | 1172 | 5 | | 1.13 | 0.93-1.38 |
| Medium Intensity‡ | 58 | 2 | 567 | 2 | | 1.00 | 0.75-1.33 |
| High Intensity¤ | 65 | 3 | 724 | 3 | | 0.86 | 0.66-1.12 |
|  |  |  |  |  | |  |  |
| Long-term >15 years: |  |  |  |  | |  |  |
| Low Intensity† | 8 | 0.3 | 64 | 0.3 | | 1.22 | 0.58-2.57 |
| Medium Intensity‡ | 10 | 0.4 | 36 | 0.2 | | 3.04 | 1.47-6.32 |
| High Intensity¤ | 7 | 0.3 | 70 | 0.3 | | 0.92 | 0.42-2.03 |
|  |  |  |  |  | |  |  |

* Matched on county of residence and birth year

^Analyses adjusted for use of hormone replacement therapy, history of rheumatoid arthritis and migraine.

† Low intensity was prescription use <25% of duration

‡ Medium intensity was prescription use >25% but <50% of duration

¤ High intensity was prescription use >50% of duration

**Appendix *Table 2: Duration and intensity of non-steroidal anti-inflammatory drug use (aspirin and non-selective non-aspirin non-steroidal anti-inflammatory drugs) among women with at least 10 years of prescription history and odds ratio of breast cancer***

| **Characteristics** | **Cases*** | | **Controls*** | | **Odds Ratio^** | | **95% Confidence Interval** |
| --- | --- | --- | --- | --- | --- | --- | --- |
|  | **N** | **%** | **N** | **%** | |  |  |
|  |  |  |  |  | |  |  |
| Never/rare | 1387 | 58 | 14372 | 60 | | 1.00 |  |
|  |  |  |  |  | |  |  |
| Short-term <10 years: |  |  |  |  | |  |  |
| Low Intensity† | 285 | 12 | 2605 | 11 | | 1.09 | 0.95-1.25 |
| Medium Intensity‡ | 195 | 8 | 1845 | 8 | | 1.04 | 0.89-1.22 |
| High Intensity¤ | 269 | 11 | 2736 | 11 | | 0.98 | 0.85-1.13 |
|  |  |  |  |  | |  |  |
| Long-term 10-<=15 years: |  |  |  |  | |  |  |
| Low Intensity† | 118 | 5 | 990 | 4 | | 1.16 | 0.94-1.42 |
| Medium Intensity‡ | 58 | 2 | 575 | 2 | | 0.98 | 0.74-1.29 |
| High Intensity¤ | 61 | 3 | 706 | 3 | | 0.83 | 0.63-1.09 |
|  |  |  |  |  | |  |  |
| Long-term >15 years: |  |  |  |  | |  |  |
| Low Intensity† | 12 | 0.5 | 75 | 0.3 | | 1.55 | 0.83-2.89 |
| Medium Intensity‡ | 9 | 0.4 | 64 | 0.3 | | 1.40 | 0.69-2.84 |
| High Intensity¤ | 5 | 0.2 | 73 | 0.3 | | 0.63 | 0.25-1.57 |
|  |  |  |  |  | |  |  |

* Matched on county of residence and birth year

^Analyses adjusted for use of hormone replacement therapy, history of rheumatoid arthritis and migraine.

† Low intensity was prescription use <25% of duration

‡ Medium intensity was prescription use >25% but <50% of duration

¤ High intensity was prescription use >50% of duration

**Appendix *Table 3: Duration and intensity of aspirin use among women with at least 10 years of prescription history and odds ratio of breast cancer***

| **Characteristics** | **Cases*** | | **Controls*** | | **Odds Ratio^** | | **95% Confidence Interval** |
| --- | --- | --- | --- | --- | --- | --- | --- |
|  | **N** | **%** | **N** | **%** | |  |  |
|  |  |  |  |  | |  |  |
| Never/rare | 2071 | 86 | 20926 | 87 | | 1.00 |  |
|  |  |  |  |  | |  |  |
| Short-term <10 years: |  |  |  |  | |  |  |
| Low Intensity† | 74 | 3 | 761 | 3 | | 0.97 | 0.76-1.25 |
| Medium Intensity‡ | 86 | 4 | 665 | 3 | | 1.29 | 1.02-1.63. |
| High Intensity¤ | 135 | 6 | 1366 | 6 | | 0.98 | 0.81-1.18 |
|  |  |  |  |  | |  |  |
| Long-term 10-<=15 years: |  |  |  |  | |  |  |
| Low Intensity† | 19 | 0.8 | 123 | 0.5 | | 1.55 | 0.94-2.53 |
| Medium Intensity‡ | 11 | 0.5 | 93 | 0.4 | | 1.15 | 0.61-2.16 |
| High Intensity¤ | 1 | 0.04 | 93 | 0.4 | | 0.10 | 0.01-0.75 |
|  |  |  |  |  | |  |  |
| Long-term >15 years: |  |  |  |  | |  |  |
| Low Intensity† | 2 | 0.08 | 4 | 0.01 | | 6.35 | 1.05-38.33 |
| Medium Intensity‡ | 0 | 0 | 5 | 0.02 | | * | * |
| High Intensity¤ | 0 | 0 | 5 | 0.02 | | * | * |
|  |  |  |  |  | |  |  |

* Matched on county of residence and birth year

^Analyses adjusted for use of hormone replacement therapy, history of rheumatoid arthritis and migraine.

† Low intensity was prescription use <25% of duration

‡ Medium intensity was prescription use >25% but <50% of duration

¤ High intensity was prescription use >50% of duration
